# Two-stage induced differentiation of OCT4^+^/Nanog^+^ stem-like cells in lung adenocarcinoma

**DOI:** 10.18632/oncotarget.11721

**Published:** 2016-08-31

**Authors:** Rong Li, Jinsu Huang, Meili Ma, Yuqing Lou, Yanwei Zhang, Lixia Wu, David W. Chang, Picheng Zhao, Qianggang Dong, Xifeng Wu, Baohui Han

**Affiliations:** ^1^ Department of Pulmonary Medicine, Shanghai Chest Hospital, Shanghai Jiao Tong University, Shanghai 200030, China; ^2^ Cancer Stem Cells Research Group, Shanghai Jiaotong University Cancer Institute, Shanghai 200032, China; ^3^ Department of Epidemiology, The University of Texas MD Anderson Cancer Center, Houston, Texas 77030, USA; ^4^ Department of Pathology and Genomic Medicine, Houston Methodist Hospital Research Institute, Houston, Texas 77030, USA

**Keywords:** cancer stem cell, differentiation, lung adenocarcinoma, vitamin D, OCT4

## Abstract

Stem-like cells in solid tumors are purported to contribute to cancer development and poor treatment outcome. The abilities to self-renew, differentiate, and resist anticancer therapies are hallmarks of these rare cells, and steering them into lineage commitment may be one strategy to curb cancer development or progression. Vitamin D is a prohormone that can alter cell growth and differentiation and may induce the differentiation cancer stem-like cells. In this study, octamer-binding transcription factor 4 (OCT4)-positive/Nanog homeobox (Nanog)- positive lung adenocarcinoma stem-like cells (LACSCs) were enriched from spheroid cultured SPC-A1 cells and differentiated by a two-stage induction (TSI) method, which involved knockdown of hypoxia-inducible factor 1-alpha (HIF1α) expression (first stage) followed by sequential induction with 1alpha,25-dihydroxyvitamin D3 (1,25(OH)_2_D_3_, VD3) and suberoylanilide hydroxamic acid (SAHA) treatment (second stage). The results showed the HIF1α-knockdowned cells displayed diminished cell invasion and clonogenic activities. Moreover, the TSI cells highly expressed tumor suppressor protein p63 (P63) and forkhead box J1 (FOXJ1) and lost stem cell characteristics, including absent expression of OCT4 and Nanog. These cells regained sensitivity to cisplatin *in vitro* while losing tumorigenic capacity and decreased tumor cell proliferation *in vivo*. Our results suggest that induced transdifferentiation of LACSCs by vitamin D and SAHA may become novel therapeutic avenue to alter tumor cell phenotypes and improve patient outcome.

**SIGNIFICANCE STATEMENT:**

The development and progression of lung cancer may involve rare population of stem-like cells that have the ability to grow, differentiate, and resist drug treatment. However, current therapeutic strategies have mostly focused on tumor characteristics and neglected the potential source of cells that may contribute to poor clinical outcome. We generated lung adenocarcinoma stem-like cells from spheroid culture and induced their transdifferentiation by a two-stage method of knocking down HIF1α expression followed by vitamin Dand suberoylanilide hydroxamic acid (VD3/SAHA) treatment. We observed the induced cells lost stem-like characteristics, regained sensitivity to cisplatin, and displayed reduced tumorigenic capacity. These findings suggest that targeting stem-like cells by reverting them to more specialized state may be an approach to treat lung cancer.

## INTRODUCTION

Lung cancer is the leading cause of cancer death for both men and women in the United States [1] and several developing countries including China. The majority (∼80%) of lung cancer is non-small cell lung cancer (NSCLC), among which adenocarcinoma is the most common histological type comprising of 30-50% of cases [2]. The existence of lung cancer stem cells, which have the ability to self-renew, differentiate, proliferate, and be highly resistant to chemoradiation treatment, have been proposed based on experimental and clinical evidence. These cells are thought to give rise to tumor cells during cancer development and are the source of recurrence and metastasis leading to poor survival [3–4].

The expression of pluripotent transcription factors, especially OCT4 and Nanog, are widely accepted as lung stem cells markers, and developmentally regulated Notch, Wnt, and Hedgehog pathways are considered as important signaling pathways which are responsible for the transformation of normal stem cells [2]. However, the biology of lung stem cell development is still not fully understood and no definitive strategies exist on how to treat lung cancer through targeting cancer stem-like cells (CSCs). Induced pluripotent stem cells are known to engage in transdifferentiation, which is defined as dedifferentiation of the primary cell and then differentiation into a new lineage [5]. During this process, the CSCs would lose stemness and become another cell type which is different from the original cell lineage from which the tumor arises [6]. This feature offers a potential therapeutic strategy to treat or cure lung cancer.

Vitamin D is a multifunctional prohormone that has been reported to induce cancer cell differentiation into a less malignant and more mature phenotype [7]. Moreover, despite the varied evidence, epidemiologic and clinical studies have suggested a possible protective role of vitamin D to reduce the incidence and progression of several kinds of cancer, including colon cancer, lung cancer, breast cancer and prostate cancer [7]. We hypothesize that vitamin D may play an important role in the transdifferentiation of lung adenocarcinoma stem-like cells (LACSCs), based on the prohormone's ability to downregulate OCT4 and Nanog [8], which are essential in the maintenance of the stemness feature in cancer cells [9–10]. So far, the differentiation effect of vitamin D3 on LACSCs has not been well-studied.

In this study, we show that through a two-stage-induction method which included the knockdown of hypoxia inducible factor 1a (HIF1a) gene expression and then sequential induction by 1alpha,25-dihydroxyvitamin D3 (1,25(OH)_2_D_3_, VD3) combined with suberoylanilide hydroxamic acid treatment (VD3/SAHA) in the second stage, the LACSCs changed to differentiated state with disappearance of stem cell markers; diminished ability to invade and clonogenic capacity *in vitro*; regained sensitivity to cisplatin; lost tumorigenic capacity and decreased tumor cell proliferation *in vivo*. We demonstrated that LACSCs can be differentiated in two stages in which VD3/SAHA play a crucial role. Our data suggest that VD3 in combination with other therapeutic agents may be candidate for treating chemoresistant LACSCs and provide a novel therapy for chemotherapy resistant lung adenocarcinoma.

## RESULTS

### Overview

**Figure 1:**
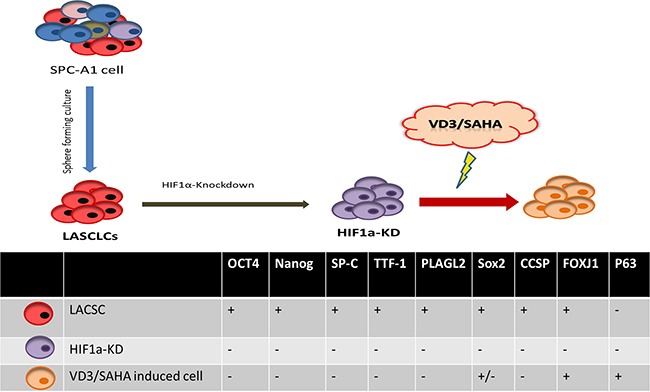
Schematic diagram of two-stage differentiation of lung adenocarcinoma stem-like cells (LACSCs) and cell surface marker expression

In this study, the differentiation of LACSCs was carried out in two phases (Figure 1). First, we stably knocked down HIF1α expression in SPC-A1 lung adenocarcinoma cells by HIF-1α shRNA lentiviral particles, which resulted in changing the stem-like cells to an intermediate differentiated state (named HIF1α-KD). In the second stage, we treated HIF1α-KD cells with vitamin D and SAHA, which transformed these cells into P63 and FOXJ1 positive differentiated cells (Figure 1). It should be noted that SPC-A1 cells expressed vitamin D receptor (VDR) (Supplementary Figure S1).

### Expression of OCT4/Nanog in LACSCs

**Figure 2:**
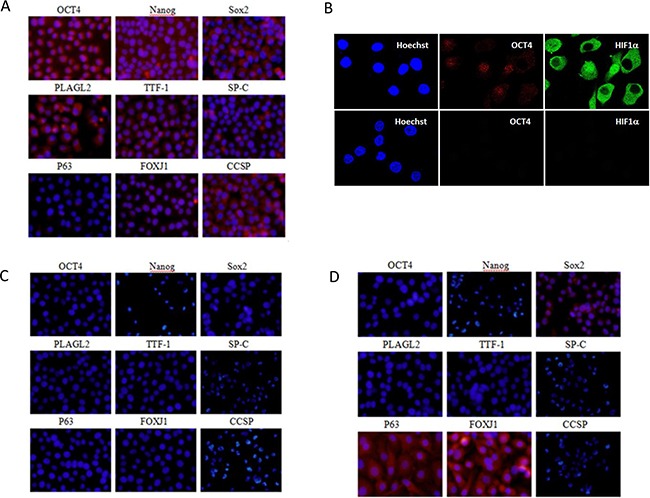
Immunofluorescent staining images of LACSC marker expression before and after each stage of differentiation **A.** Cell marker expression of OCT4, Nanog, Sox2, PLAGL2, TTF-1, SP-C, P63, FOXJ1, and CCSP protein expression in parental LACSCs (SPC-A1 spheroid cells); **B.** OCT4 and HIF1α expression and Hoechst nuclear staining in LACSCs (upper panels) and HIF1α-KD cells (lower panels); **C.** cell marker expression in HIF1α-KD cells; **D.** marker expression in HIF1α-KD-SAHA/VD3 induced cells. All images were taken at 200x magnification. Figure 2B images were enlarged digitally to visualize individual cells more clearly for protein subcellular localization.

We enriched LACSCs from SPC-A1 cells by spheroid culture methodology, which has been widely used [11–12]. Immunofluorescence analysis demonstrated the spheroid cultured SPC-A1 cells positively express OCT4 and Nanog (Figure 2A), two of the signature embryonic stem cell transcription factors. The results of immunofluorescence analysis also showed that the LACSCs positively expressed surfactant protein C (SP-C), thyroid transcription factor-1 (TTF-1), pleiomorphic adenoma gene-like 2 (PLAGL2), SRY (sex determining region Y)-box 2 (Sox2), Clara cell secretory protein (CCSP), and forkhead box protein (FOXJ1). P63 expression was negative in these cells (Figure 2A).

### Expression of cell markers on HIF1α-KD cells

We stably depleted HIF1α expression in SPC-A1 lung adenocarcinoma cells by HIF-1α shRNA lentiviral particles. To verify the specificity of knockdown, we measured the endogenous expression of *HIF1A* gene in the HIF1α-KD cells and found significantly reduced expression whereas *HIF2A*, which shares high sequence homology as *HIF1A*, was not affected (Supplementary Figure S2). Imaging by laser confocal fluorescence microscopy demonstrated the HIF1α-KD cells negatively expressed HIF1α confirming the effective repression of this gene product (Figure 2B). In addition, the knockdown cells also lacked the expression of OCT4 (Figure 2B). The expression of cell markers on HIF1α-KD cells was substantially altered (Figure 2C). The result of immunofluorescence analysis showed that HIF1α-KD cells negatively expressed stem cell markers and the other cell markers, including OCT4, Nanog, SP-C, TTF-1, PLAGL2, Sox2, CCSP, FOXJ1 and P63.

### Invasion activity

**Figure 3:**
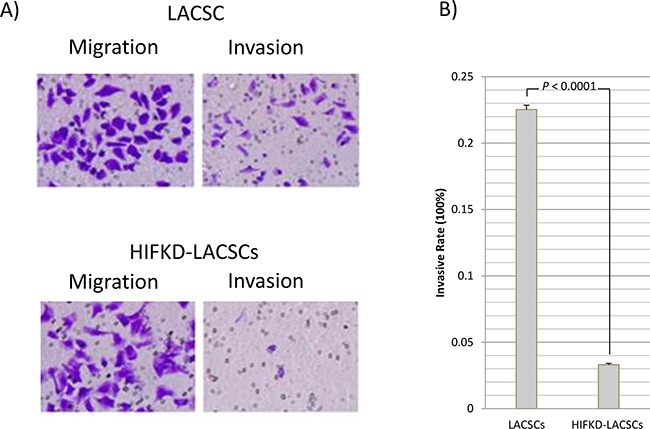
Migration and invasion activities of LACSCs and HIF1α-KD cells **A.** Crystal violet staining of LACSCs and HIF1α-KD cells showing migration (left) and invasion (right) activities. **B.** Invasion rate of LACSCs and HIF1α-KD cells. Results are averages of 3 replicates.

The lack of expression of stem-cell markers in HIF1α-KD LACSCs suggest these cells may possess altered cellular characteristics compared to LACSCs, so we tested the cells in various phenotypic assays. The HIF1α-KD LACSCs displayed decreased invasion activity compared to LACSCSs but not migration activity (Figure 3A). The invasive rate was 22.5±0.3% for LACSCs compared to 3.31±0.1% for HIF1α-KD LACSCs (Figure 3B) (*p*<0.0001).

### Colony formation

**Figure 4:**
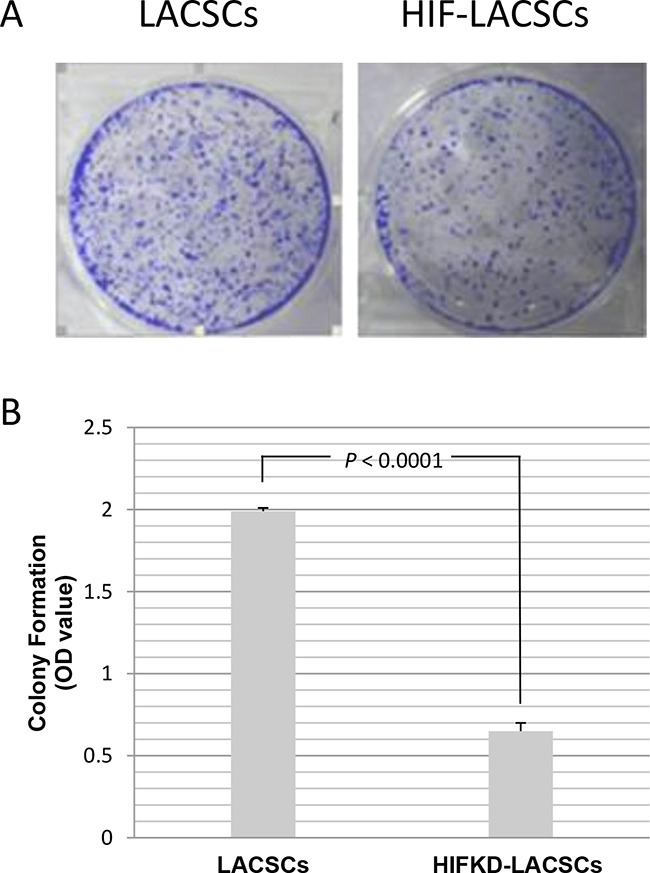
Growth of LACSCs and HIF1α-KD induced cells as measured by colony formation assay Cells were stained with crystal violet and then stain dissolved in 3% acetic acid and measured for absorbance at 570 nm. **A.** The crystal violet stained plates of LACSCs and HIF1α-KD cells in colony formation assay; **B.** Relative growth of LACSCs and HIF1α-KD cells. Results are averages of 3 replicates.

Colony formation assay showed that the number of foci of HIF1α-KD LACSC were significantly less than that of LACSCs (Figure 4A). Measurement of crystal violet staining confirmed the results showing the optical density (OD) of LACSCs was 1.99±0.02, while the OD was 0.65±0.05 for HIF1α-KD LACSCs (Figure 4B) (*P*<0.0001).

### Expression of cell markers on VD3/SAHA induced cells

Next, we subjected the HIF1α-KD cells to VD3 and SAHA treatment and then examined the expression of previously tested cell markers. Immunostaining of the induced cells showed P63 and FOXJ1 were positive, while OCT4, Nanog, SP-C, TTF-1, PLAGL2, and CCSP were all negative. Interestingly, Sox2 was reexpressed although weakly (Figure 2D).

### Sensitivity to chemotherapy

**Figure 5:**
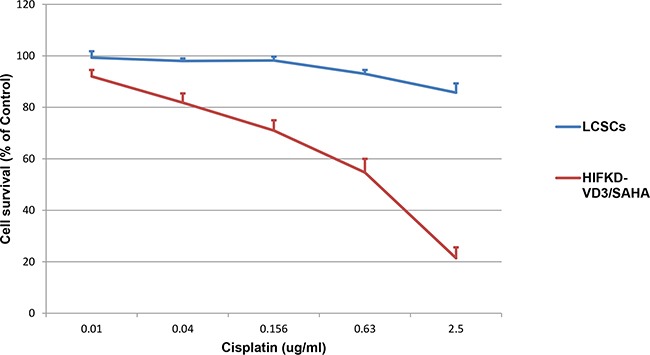
HIF1α-KD-VD3/SAHA induced cells were more sensitive to cytotoxic effect of cisplatin compared to parental LACSCs Results are averages of 3 replicates.

To see whether LACSCs and HIF1α-KD-VD3/SAHA induced cells showed differential sensitivity to chemotherapeutic agent cisplatin, we tested the cells in cytotoxic assay. Figure 5 showed that LACSCs were highly resistant to cisplatin, while HIF1α-KD -VD3/SAHA induced cells were much more sensitive to cisplatin. The 50% inhibitory concentration (IC50) of cisplatin in HIF1α-KD -VD3/SAHA induced cells was 0.51±0.03μg/ml, which is significantly lower than the peak plasma concentrations of cisplatin (about 4 μg/ml) during standard chemotherapeutic treatment.

### Tumorigenicity in vivo

**Figure 6:**
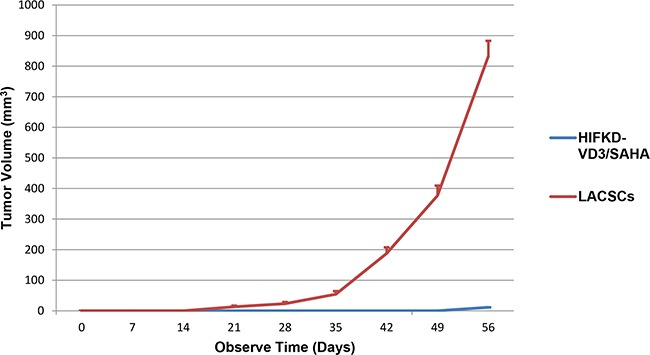
Tumorigenic effect of un-induced and differentiated LACSCs *in vivo* Xenograft tumor growth of LACSC spheroid cells and HIF1α-KD-VD3/SAHA induced cells were measured in NOD-SCID immunodeficient mice. Results are shown as averages of two independent experiments.

Differentiation of LACSCs should not only improve the sensitivity to chemotherapy, but also reduce their tumorigenicity. To determine whether the tumor growth of HIF1α-KD-VD3/SAHA induced cells decreased*in vivo*, we compared the tumorigenicity of HIF1α-KD-VD3/SAHA induced cells with LACSCs in NOD-SCID mice in xenograft experiments. The results for two independent experiments showed that the tumorigenicity rates were both 100% for LACSCs, while it was 0% and 50% for HIF1α-KD-VD3/SAHA induced cells. Therefore, the average tumorigenicity rate for HIF1α-KD-VD3/SAHA induced cells was 25%. Figure 6 showed that the tumor volumes of LACSCs were much larger than HIF1α-KD-VD3/SAHA induced cells during the follow-up period of growth. Tumor volume of LACSCs and the induced cells at the 56^th^ day were 905.2±13.5mm^3^ *vs* 0.5±0.04 mm^3^, respectively, *p*<0.0001 (Supplementary Table S1). This demonstrates that the sequential differentiation of LACSCs could significantly reduce their tumorigenic potential *in vivo*.

## DISCUSSION

In this study, we demonstrated that LACSCs can be induced to differentiate into squamous-like cells by a two-stage method. This transdifferentation was characterized by the reacquirement of proximate bronchial epithelium markers (i.e., Sox2, P63 and FOXJ1) and the disappearance of lineage markers for distal respiratory cells, including SP-C, CCSP, TTF-1 and PLAGL2. The phenotypic switch was accompanied with an increased sensitivity to cisplatin. Notably, these induced cells also lost the ability to invade *in vitro* and become much less tumorigenic *in vivo*. The data provided herein suggested that these putative LACSCs are bipotential cells capable of differentiating into two distinct lineages of lung airway epithelial cells. The reduced capacities of metastasis and tumorigenesis after their transdifferentiation may provide new avenue in the treatment of these highly aggressive cells.

It is widely accepted that the CSC-like cells expressing OCT4 within the tumor are responsible for disease progression and poor patient survival [13–14]. In lung cancer, these stem-like cells can be enriched by using of sphere-forming assay and were positive for OCT4 and Nanog expression [7, 15]. Some researchers reported that OCT4 expression in solid tumors correlated with hypoxia-induced HIF1A expression [16], while others demonstrated that HIF2A plays a critical role in the transcriptional regulation of this pluripotent gene [17]. To evaluate the potential role of HIF1A in our cell model, we depleted its expression with specific shRNA lentiviral particles. The results showed that knockdown of HIF-1A significantly reduced the expression of OCT4, demonstrating that OCT4 expression can be regulated by HIF1A in lung cancer cells. Interestingly, we also found that accompanied by the loss of OCT4 expression, the LACSC-derived cells lost the cancer hallmarks associated with stemness, as shown by decreased invasion, migration and clonogenic capacity *in vitro*. Moreover, phenotypic analysis showed that these OCT4^−^ HIFα-KD cells lost the expression of tested markers, including those associated with the proximate and distal respiratory epithelium of the lung. Based on these findings, we considered HIFα-KD cells as intermediate cells.

Previously, we observed that the serum level of VD3 in lung adenocarcinoma patients was remarkably reduced when compared with healthy subjects and other histological types of lung cancer [18]. VD3 is a multifunctional hormone that has been reported to induce cancer cell differentiation into a less malignant and more mature phenotype [7]. We tested the potential actions of VD3 in our cell model and found that compound alone did not induce any observable phenotypic effects on LACSCs and HIF1α-KD cells, such as cell growth and marker expression, etc. (data not shown). However, by searching the literature, we found VD3 was able to regulate *P63* gene expression via the transcriptional repressor DEC1 [19], which is positively controlled by VD3 receptor (VDR) and negatively regulated by HDAC1 [20]. Accordingly, we tried to assess the possible role of VD3 and SAHA, a small molecular inhibitor of HDAC1, in combination. We observed for the first time that VD3/SAHA in combination did play a role in the transdifferentiation of HIF1α-KD intermediate cells but not LACSCs, and induced HIF1α-KD cells to express Sox2, P63 and FOXJ1, the markers that are normally expressed in the basal cells of the adult human lung. In contrast, the HIF1α-KD cells after this treatment remained negative for the expression of distal cell markers, which are normally present in Clara cells and type II pneumocytes.

The re-expression of FOXJ1 and to lesser extent Sox2 after VD3/SAHA induction is notable. FOXJ1 belongs to the Foxhead box (FOX) family of transcription factors, which regulate a variety of biological process involving cell fate determination [21]. *FOXJ1* along with other FOX-related genes may be negatively regulated targets of Nanog in embryonic stem cells. In ovarian cancer, stable knockdown of *NANOG* led to decreased cell proliferation, migration, and invasion, which were accompanied by increased expression of E-cadherin and FOX-related genes including *FOXJ1* [22], results consistent with our findings. The expression of Sox2, albeit weakly, in the transdifferentiated cells is somewhat perplexing. However, it is known that varying levels of Sox2 can affect fate of embryonic stem cell differentiation, and that expression of Sox2 can inhibit differentiation of mesodermal germ layer but promote differentiation into the neural/ectodermal lineage [23] indicating both positive and negative regulatory roles.

There are some limitations in our study. First, we have only shown the results for one lung cancer cell line, SPC-1A cells, which is the first lung adenocarcinoma cell line established in China [24]. We have evaluated two additional lung adenocarcinoma cell lines (A549, and PC3) and found that they were not suitable for our study because these cells failed to express proximate lineage markers. SPC-A1 cells are wild-type for both *KRAS* and *EGFR* [25, 26], unlike A549 cells which are mutant for *KRAS* and PC3 cells mutant for *EGFR*. Therefore, it is possible that transdifferentiation of lung cancer cells might be affected by different oncogenic mutation status. We are preparing to further study the phenomena of LACSC (spheroid) enrichment and their transdifferentiation under different driving gene mutations and to assess whether these events are unique to SPC-A1 cells. Second, although the levels of SAHA and VD3 used for our *ex vivo* experiments are similar to previous studies [27, 28], the amount used might be in excess of physiological or pharmacological range. Additional preclinical studies are needed to determine the appropriate pharmacologic VD3/SAHA levels to revert the oncogenic phenotype without inducing overt toxicity.

Taken together, although the exact mechanisms of action for this two-stage induction of LACSC transdifferentiation remain unclear, the results described above may bring prospects for potential clinical application in the future. Chemotherapy occupies important position in the clinical treatment of lung cancer in which cisplatin is internationally recognized as first-line therapy, but its effect on survival is limited and the development of drug resistance is common. The presence of CSCs in tumors may be one of the underlying causes for recurrence and treatment failure. Researchers have reported that residual cancer cells after chemotherapy have stem-like characteristics including OCT4 expression, indicating that most chemotherapeutic regimens kill sensitive tumor cells but enrich for resistant CSCs thus leading to eventual recurrence or progression. Therefore, restoring drug sensitivity of lung CSCs is paramount to eradicate residual tumor cells and to improve patient outcome and survival. The results obtained in this study suggest that induced transdifferentiation of CSCs by vitamin D and other agents such as SAHA may become an important strategy to decrease tumor recurrence and resistance. Further clinical and functional studies are warranted to validate these findings as well as to define the molecular pathways of stem cell regulation during lung cancer development and treatment.

## MATERIALS AND METHODS

### Cell culture

SPC-A1 cells, purchased from Cell Bank of Shanghai Institute of Life Science, were cultured to spheroids to enrich stem cells. Fetal bovine serum (FBS, PAA Laboratories GmbH, Germany), DMEM medium (GIBCO, San Diego, CA), DMEM/F12 medium (HyClone, Logan, Utah) were used in the research. In sphere-forming cell culture, B27 supplement (50x) (Gibco), Heparin (Heparin, Na Salt) (Sigma-Aldrich, St. Louis, MO), Basic fibroblast growth factor (Peprotec, Rocky Hill, NJ) and epidermal growth factor (Peprotec) were used. The SPC-A1 cell concentration was adjusted to 2 × 10^4^/ml by the 1xCSC medium, which contained DMEM/F12 medium, 20 ng/ml EGF, 20 ng/ml bFGF, 4μg/ml heparin and 1x B27. The cells (500μl/well) were seeded in 24-well ultra-low absorption plates (Corning Company, cat No.3473). At day 3, 5, and 7, fresh 10xCSC medium (50μl/well) was added to each well.

### Antibodies and reagents for differentiation

Most immunofluorescence antibodies were purchased from Santa Cruz (Dallas, TX), including rabbit anti-HIF1 and anti-OCT4 antibodies OCT4 monoclonal mouse anti-human antibody, mouse monoclonal anti-human SOX2 antibody, mouse anti-human FOXJ1 monoclonal antibody, mouse anti-human P63 monoclonal antibody, goat anti-human Nanog polyclonal antibody, goat anti-human CCSP polyclonal antibody, rabbit polyclonal anti-human SP-C antibody, mouse anti-human VDR antibody, and secondary rhodamine-labeled and fluorescein-labeled donkey anti-mouse, anti-goat, and anti-rabbit IgG antibodies. TRITC-labeled donkey anti-rabbit IgG antibody was purchased from Thermo Fisher Scientific (Waltham, MA). Several chemicals were used to induce stem cell differentiation, including 1,25(OH)_2_D_3_ (VD3), purchased from Sigma and Suberoylanilide hydroxamic acid (SAHA), purchased from Selleck (Houston, TX). HIF-1α shRNA lentiviral particles sc-35561-v was bought from Santa Cruz Biotechnology (Dallas, TX).

### HIF1α knockdown and identification

SPC-A1 spheroid cells were seeded at 3 × 10^5^ cells per well (1 ml culture medium) in a 6-well plate and cultured overnight, and then were transduced with HIF-1α shRNA lentiviral particles (sc-35561-V, Santa Cruz Biotechnology) 30μl/well and cultured for 2 days. Puromycin (concentration) was used to select cells.

### Immunofluorescent staining of OCT4, HIF1α, and VDR

Cells (1× 10^5^ cells, 500μl medium) were seeded in Millicell Side chamber (Millipore Corporation). After overnight culture, the supernatant was discarded, and 100μl Cytofix fixation buffer was added to the cells and fixed for 30 min. The supernatant was discarded again and then washed with Perm/Wash buffer. After washing two times 100μl each with Perm/Wash buffer, the cells were treated with 2μl of anti-OCT4, -HIF1α, or -VDR antibody (1:50 dilution by CSC medium) or PBS alone as negative control and incubated at 4°C overnight. Next day, cells were washed by PBS three times to remove unbound antibodies, then 1μl fluorescein anti-IgG secondary antibody (1:100 dilution) was added in the dark and incubated at room temperature for 1 h. Cells were counter-stained with Hoechest 33342 (1:50 dilution) and images were captured by laser scanning confocal microscopy (Olympus corporation, FluoView FV1000).

### Cell differentiation

HIF1α knockdown (HIF1α-KD) cells and SPC-A1 spheroid cells were used for the induction experiments separately using the same culture conditions. A total of 1 × 10^5^ cells/well of SPC-A1 spheroid cells or HIF1α-KD cells were cultured in 24-well plates overnight and then 40 μM of SAHA and 2 μM of VD3 were added to each well for 24h. The same culture conditions were used for control cells, which were left untreated. After induction of differentiation, cells were collected for downstream *in vitro* and vivo characterizations and phenotypic assays.

### Isolation of cancer stem-like cells and identification

#### Spheroids culture

SPC-A1 cells were titrated to 2 × 10^4^/ml in CSC medium, which contained DMEM/F12 medium, 20 ng/ml epidermal growth factor (EGF, Peprotec Inc., Princeton, NJ), 20 ng/ml basic fibroblast growth factor (bFGF, Peprotec Inc., Princeton, NJ), 4μg/ml heparin (Sigma Chemical Co., Munich, Germany) and 1x B27 (Gibco, Invitrogen, UK). The cells (500μl/well) were seeded in 24-hole ultra-low absorption plates (Corning Inc., Corning, NY). 10x CSC medium (50 μl/well) was added to each well at day 3, 5, and 7. Cells were collected at day 8 and then centrifuged for 2 min. The spheroid cells were resuspended in 200μl trypsin and digested for 3 minutes. Reaction was quenched by adding 800μl serum-containing medium and then living cells were counted by using Countstar automatic cell counter (Shanghai Alit companies, China).

### Expression of cell markers by immunofluorescent microscopy

Cells were seeded in Costar 24-well plates (Corning Inc.) with coverslips and cultured overnight. Next day, the cells were fixed by 4% paraformaldehyde for 30 minutes, and then washed 3 times with phosphate buffered saline (PBS) and permeabilized with Triton X-100. Primary antibodies were added to cells against OCT4, Nanog, Sox2, PLAGL2, TTF-1, SP-C, P63, FOXJ1, CCSP (1:50 dilution), and incubated overnight at 4°C. PBS was added instead of primary antibody as negative control. The following day, secondary antibody (1:100 dilution) was added to the cells and then incubated in the dark at room temperature for 1 h. Cells were counterstained with Hoechest 33342 (1:40 dilution) and imaged by Olympus IX51 fluorescence microscope (Tokyo, Japan).

### Transwell migration/invasion assay

Transwell chambers (Corning Inc., Corning, NY) were filled with 50μl of Matrigel medium (Sigma Chemical Co., Munich, Germany) diluted with cold DF12 medium without serum. Then the transwell chambers were packaged with (invasion assay) or without (migration assay) Matrigel and plated with 5 × 10^4^ cells in 200 ul serum-free DMEM medium. The chambers were inserted into 24-well plates containing 750μl 10% FCS-DMEM medium per well. The cells were cultured for 24 h at 37°C in a 5% CO2 incubator. Cells were fixed with 4% paraformaldehyde for 30min and stained with 500 μl crystal violet for 10 min. Images of cell were taken under phase contrast microscope and counted. Cell invasion rate (%) = (no. of invaded cells / no. of migrated cells) × 100%.

### Clonogenic assay

Clonogenic assay was performed as previously described [25] with slight modifications. Briefly, cells (2 × 10^2^ cells/well) were seeded in 6-well plates, which contain 2ml/well 10% FBS-DMEM medium, and cultured at 37°C for 2 weeks. Cells were fixed with 4% paraformaldehyde and then stained with 500 μl crystal violet (0.5% w/v) [24]. Colonies from 10 separate fields were counted using a microscope. Clonogenic ability was detected by crystal violet staining. To determine the relative number of clones, 3% acetic acid was used to elute the crystal violet and the absorbance was detected at 570 nm wavelength light in a spectrophotometer (Shanghai Mapada Instruments Co., Ltd).

### Microculture cytotoxicity assay

A total of 500 cells/well in 100 μl of 10% FBS-DMEM medium were plated in 96-well plates and cultured overnight. Next day, cells were exposed to chemotherapeutic agents (Cisplatin at 0.01 ug/ml∼2.5ug/ml concentrations) or medium as control and cultured for 6 days. For each condition, 6 replicates were included in the experiment. Cell survival was measured using the colorimetric CCK-8 assay Sigma Chemical Co., Munich, Germany) at 492 nm wavelength. Cell counts were determined from the standard curve of the cell number and absorbance graph. Cell viability = (no. of viable cells in the treatment group / no. of cells in the untreated group) × 100%.

### Real-time PCR assay

SPC-A1 spheroid cells were seeded at 3 × 10^5^ cells per well in 6-well plates and cultured overnight for mock and transduction with HIF-1α shRNA lentiviral particles under Puromycin selection. After 2 days of selection, total RNA was extracted and reverse transcribed using total mRNA extraction and cDNA synthesis kits (Sigma-Aldrich, St. Louis, MO) according to the manufacturer's instructions. The primers for HIF1A, HIF-2A and GAPDH (normalization control) were designed and purchased from Shanghai Sunteam Biotech Co., Ltd. The primers used were: HIF-1α forward: 5′-GAAAGCGCAAGTCTTCAAAG-3′; HIF-1α reverse: 5′-TGGGTAGGA-GATGGAGATGC-3′; HIF-2α forward: 5‘-TCTGAAAACGAGT CCGAAGCC-3‘; HIF-2α reverse: 5‘-GGTCGCAGGGATGAGTGAAGT-3‘; GAPDH forward: CGGAGTCAAC GGATTTGG TCGTAT; and GAPDH reverse: AGCCTTCTCC ATGGTGGTGAAGAC. Real-time PCR was carried out using ABI7500 Sequence Detector System (Applied Biosystems, USA).

### Xenograft assay

All animal experiments were approved by the National Natural Science Foundation of China (NO: 81101770). Mice were manipulated and housed according to protocols approved by the Shanghai Medical Experimental Animal Care Commission. Eight-weeks-old-female NOD-SCID mice with 18-22 g weight were injected subcutaneously with 5 × 10^4^ of spheroid SPC-A1 cells cultured for 7 days on the right side of the abdominal wall and the same number of differentiated cells (SAHA/VD3 treated as described above and also cultured for 7 days) on the left side of the abdomen. The day that cells were seeded was marked as Day 0. Animals were observed and measured for tumor growth every week. Calipers were used to measure maximum tumor diameter (a) and its vertical diameter (b). Measurements are accurate to millimeter. Tumor volumes were calculated by the formula: V = 0.5 × ab^2^. Total observation period is 7 weeks. These experiments were repeated twice with six NOD-SCID mice participated each time.

### Statistical analysis

Results were shown as mean±SEM. Student *t* test or ANOVA was used to assess the significance of differences between means of test groups. Differences were considered statistically significant at *P*<0.05 two tailed. All statistics were carried out using SPSS 11.0 software.

## SUPPLEMENTARY MATERIALS FIGURES AND TABLE




