# Supplementary material for: Two-stage induced differentiation of OCT4+/Nanog+ stem-like cells in lung adenocarcinoma
Source: Oncotarget. 2016 Aug 31;7(42):68360–70. doi: 10.18632/oncotarget.11721 (PMC5356561; doi:10.18632/oncotarget.11721)
Supplement: Supplementary file 1 [file oncotarget-07-68360-s001.pdf]

Two-stage induced differentiation of OCT4^{+}/Nanog^{+} stem-like cells in lung adenocarcinoma

SUPPLEMENTARY FIGURES AND TABLE

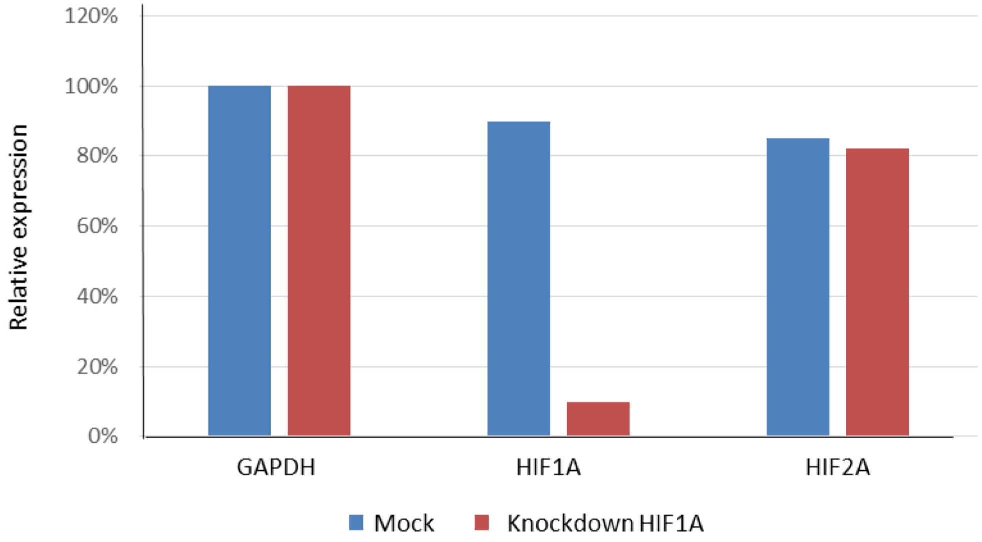

Supplementary Figure S1: Vitamin D receptor is expressed in SPC-A1 cells. Vitamin D receptor expression and Hoechst nuclear staining in SPC-A1 cells; probed with anti-VDR antibody (upper panels) and negative control (lower panels). Image taken at 200x magnification.

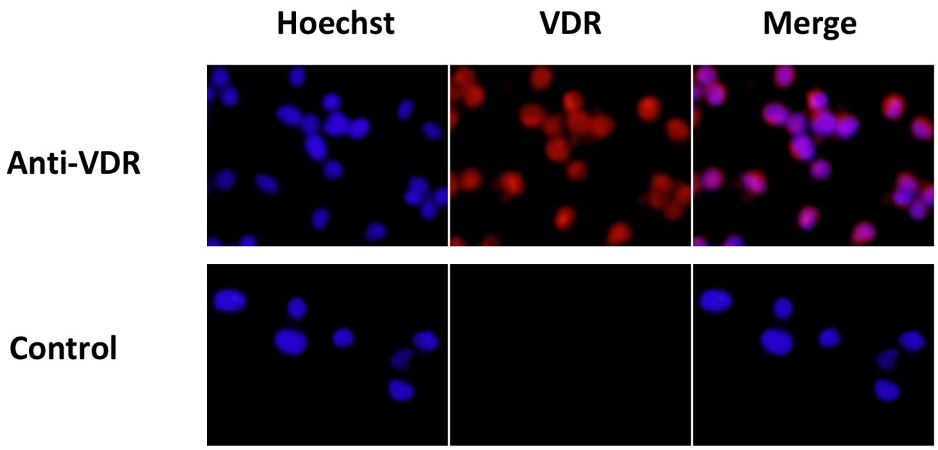

Supplementary Figure S2: Endogenous expression of *HIF1A* and *HIF2A* in mock and HIF1α shRNA treated SPC-A1 spheroid cells. Expression was measured by real-time PCR in duplicates and normalized to housekeeping gene *GAPDH* expression.

Supplementary Table S1: Xenograft experiment tumor volume data.

See Supplementary File 1
