# Supplementary material for: Two-stage induced differentiation of OCT4+/Nanog+ stem-like cells in lung adenocarcinoma
Source: Oncotarget. 2016 Aug 31;7(42):68360–70. doi: 10.18632/oncotarget.11721 (PMC5356561; doi:10.18632/oncotarget.11721)
Supplement: Supplementary file 2 [file oncotarget-07-68360-s002.docx]

| **Supplemental Table 1. Xenograft Experiment Tumor Volume Data** | | | | | | | | | | | | |
| --- | --- | --- | --- | --- | --- | --- | --- | --- | --- | --- | --- | --- |
| **Xenograft Experiment Trial One** | | | | | | | | | | | | |
| Day | MouseNo.1 | | MouseNo.2 | | MouseNo.3 | | MouseNo.4 | | MouseNo.5 | | MouseNo.6 | |
|  | LS (mm^3^) | RS (mm^3^) | LS (mm^3^) | RS (mm^3^) | LS (mm^3^) | RS (mm^3^) | LS (mm^3^) | RS (mm^3^) | LS (mm^3^) | RS (mm^3^) | LS (mm^3^) | RS (mm^3^) |
| 0 | 0 | 0 | 0 | 0 | 0 | 0 | 0 | 0 | 0 | 0 | 0 | 0 |
| 7 | 0 | 0 | 0 | 0 | 0 | 0 | 0 | 0 | 0 | 0 | 0 | 0 |
| 14 | 0 | 0 | 0 | 0 | 0 | 0 | 0 | 0 | 0 | 0 | 0 | 0 |
| 21 | 0 | 9.8 | 0 | 15.9 | 0 | 15.3 | 0 | 8.9 | 0 | 12.4 | 0 | 12.5 |
| 28 | 0 | 49.5 | 0 | 16 | 0 | 58.9 | 0 | 9 | 0 | 12.5 | 0 | 12.6 |
| 35 | 0 | 52.3 | 0 | 53.2 | 0 | 69.2 | 0 | 33.1 | 0 | 53.1 | 0 | 54.2 |
| 42 | 0 | 175.8 | 0 | 171.7 | 0 | 215.7 | 0 | 160.9 | 0 | 187.5 | 0 | 213.9 |
| 49 | 0 | 355.5 | 0 | 381.1 | 0 | 390.8 | 0 | 354.4 | 0 | 339.9 | 0 | 388.3 |
| 56 | 0 | 832.1 | 0 | 835.1 | 0 | 831.5 | 0 | 802.5 | 0 | 773.4 | 0 | 830.2 |
| **Xenograft Experiment Trial Two** | | | | | | | | | | | | |
| Day | MouseNo.1 | | MouseNo.2 | | MouseNo.3 | | MouseNo.4 | | MouseNo.5 | | MouseNo.6 | |
|  | LS (mm^3^) | RS (mm^3^) | LS (mm^3^) | RS (mm^3^) | LS (mm^3^) | RS (mm^3^) | LS (mm^3^) | RS (mm^3^) | LS (mm^3^) | RS (mm^3^) | LS (mm^3^) | RS (mm^3^) |
| 0 | 0 | 0 | 0 | 0 | 0 | 0 | 0 | 0 | 0 | 0 | 0 | 0 |
| 7 | 0 | 0 | 0 | 0 | 0 | 0 | 0 | 0 | 0 | 0 | 0 | 0 |
| 14 | 0 | 0 | 0 | 0 | 0 | 0 | 0 | 0 | 0 | 0 | 0 | 0 |
| 21 | 0 | 18.1 | 0 | 16.3 | 0 | 14.9 | 0 | 8.9 | 0 | 7.8 | 0 | 16.9 |
| 28 | 0 | 61 | 0 | 16.4 | 0 | 14.9 | 0 | 9 | 0 | 7.8 | 0 | 17 |
| 35 | 0 | 65.7 | 0 | 53.9 | 0 | 54.1 | 0 | 50.4 | 0 | 42 | 0 | 68.5 |
| 42 | 0 | 224.4 | 0 | 177.4 | 0 | 188.5 | 0 | 188.9 | 0 | 170.5 | 0 | 176.5 |
| 49 | 0 | 405.3 | 0 | 341.1 | 0 | 400.5 | 0 | 421.1 | 0 | 326.5 | 0 | 418.3 |
| 56 | 11.5 | 835.7 | 0 | 852.6 | 11.1 | 950.6 | 0 | 870.6 | 0 | 746.1 | 11.3 | 829.3 |
| LS- left side; RS- right side | | |  |  |  |  |  |  |  |  |  |  |
